# In Vitro Analysis of PMEPA1 Upregulation in Mesenchymal Stem Cells Induced by Prostate Cancer Cells

**DOI:** 10.3390/ijms26136223

**Published:** 2025-06-27

**Authors:** Aigul R. Rakhmatullina, Mariya A. Zolotykh, Yuliya V. Filina, Aisylu R. Sagdeeva, Elvira V. Rozhina, Aida G. Gabdoulkhakova, Eugenia A. Boulygina, Regina R. Miftakhova

**Affiliations:** Institute of Fundamental Medicine and Biology, Kazan Federal University, 420008 Kazan, Russia

**Keywords:** PMEPA1, TGF-β, mesenchymal stem cells, prostate cancer, tumor microenvironment

## Abstract

Isoforms of prostate transmembrane protein, androgen induced 1 (PMEPA1), are regulated either by TGF-beta or AR activation and provide negative loop-regulation of these signaling pathways. High levels of PMEPA1 protein have been observed in various tumor types, including prostate, bladder, colorectal cancers, and glioblastoma. Direct oncogenic role of PMEPA1 in hepatocellular carcinoma has been recently shown on an animal model. New studies also indicate an upregulation of PMEPA1 in tumor-associated immune and stromal cells; however, its specific role in tumor stromal cells remains largely unexplored. In our previous research, we developed a cancer-stroma sphere (CSS) model that integrates tumor cells with mesenchymal stem cells (MSCs). Evaluations of chemotherapy and CAR-T therapies on CSSs have demonstrated that this model closely mimics in vivo data regarding cytotoxicity and adverse effects of therapy. In the present study, we reveal that *PMEPA1* is significantly overexpressed in MSCs within the CSS. Moreover, this overexpression has been induced under short-term co-culture conditions. Among the five isoforms of PMEPA1, PMEPA1a and PMEPA1b isoforms have been detected in MSCs. These findings underscore the potential role of PMEPA1 in the tumor microenvironment modulation by MSCs.

## 1. Introduction

TGF-beta signaling pathway is an important regulator of tumor progression. The effect of TGF-β activation shifts from a suppressive to a tumor-promoting, depending on the type of tumor and the stage of disease: TGF- β inhibits the proliferation of tumor cells, but facilitates epithelial–mesenchymal transition and the metastasis of tumor cells. The TGF- β family includes TGF-β1, TGF-β2, TGF-β3, activins and bone morphogenetic proteins (BMPs).

*PMEPA1* acts as a target gene and regulator of the TGF- β signaling pathway [1]. Initially the gene was characterized as an androgen-responsive factor on prostate LNCaP cells [2]. In three years, GeneChip expression microarray analysis of VACO330 colon cancer cells helped to identify *PMEPA1* as a TGF- β -induced gene [3]. Furthermore, *PMEPA1* transcription has been described as an early event of TGF-beta signaling activation [4]. PMEPA1 contains PY and SH3 motifs, SIM- and WW-binding domains, that allow protein binding to SMAD2-, SMAD3-, AR- and HECT-type E3 ubiquitin ligases [4,5]. PMEPA1 and SMAD2 interaction inhibits SMAD2–SMAD4 complex formation, which prevents the initiation of transcription of TGF-β-target genes, realizing the negative loop of TGF-β control. PMEPA1 can influence TGF-β-induced responses but does not interfere with BMP-signaling [4].

A direct oncogenic activity of PMEPA1 was demonstrated recently through the hyperexpression of PMEPA1: mice were i.v. injected with transposon-based vectors carrying the *MYC* and *PMEPA1* genes—60% of animals developed hepatocellular carcinoma, whereas mice that received only *MYC* did not develop tumors [6]. The supportive role of PMEPA1 was demonstrated on a prostate cancer model, where low levels of PMEPA1 correlated with low metastasis-free survival [7]. Authors illustrate that PMEPA1 can interact with negative regulators of TGF-beta HECT E3 ubiquitin ligases (SMURF2, NEDD4-2, AIP-2) which may lead to a decrease in TGF-beta level and block its pro-metastatic function.

Five isoforms of PMEPA have been identified: a, b, c, d, and e [5]. Protein isoforms vary in size (from 237 AA for c isoform to 344 AA for e isoform), structure (c isoform is lacking extracellular domain), expression pattern and functional impact. Thus, a, c and d isoforms are believed to be TGF-β-responsive, b and e isoforms are activated by androgens, and a and d isoforms can inhibit TGF-β signaling, whereas c isoform is lacking that ability [8].

In addition to tumor cells, PMEPA1 expression has been demonstrated for mesenchymal stem (MSCs), endothelial and immune cells [6,9]. Alteration of PMEPA1 level and its role in tumor progression still needs to be elucidated.

MSCs are multipotent cells that in physiological conditions maintain the structure and function of connective tissue and control the process of tissue regeneration at damage sites [10]. Malignant tumors secrete bioactive molecules that closely resemble those released by injured tissues, thereby attracting MSCs to the tumor site. The migration and infiltration of MSCs into the primary regions of tumor growth, coupled with their interactions with tumor cells and the surrounding tumor microenvironment, can lead to the reprogramming of “normal” MSCs into tumor-associated TA-MSCs or their differentiation into tumor-associated fibroblasts (TAFs) [11]. Among the various stromal cells present within the tumor microenvironment, TAFs are particularly significant in promoting tumor progression.

The amount of MSCs in a tumor varies depending on the type and stage of the disease. For example, in prostate cancer samples, MSC content ranges from 0.01% to 1.1%, in lung cancer samples from 0.007% to 4.49%, and in glioma samples from 0.7% to 19.5% [12,13,14]. High MSC content in tumors is associated with poor disease prognosis.

The role of PMEPA1 expression in MSC remains poorly understood. Furthermore, there is a lack of information regarding the specific isoforms of PMEPA1 that are expressed in MSCs, as well as the modulation of their expression levels in response to various stimuli. This gap in knowledge highlights the need for further investigation into the functional significance of PMEPA1 in MSCs and its potential implications in the tumor microenvironment modulation.

## 2. Results

### 2.1. PMEPA1 Is Overexpressed in CSS-Derived MSCs

**Figure 1:**
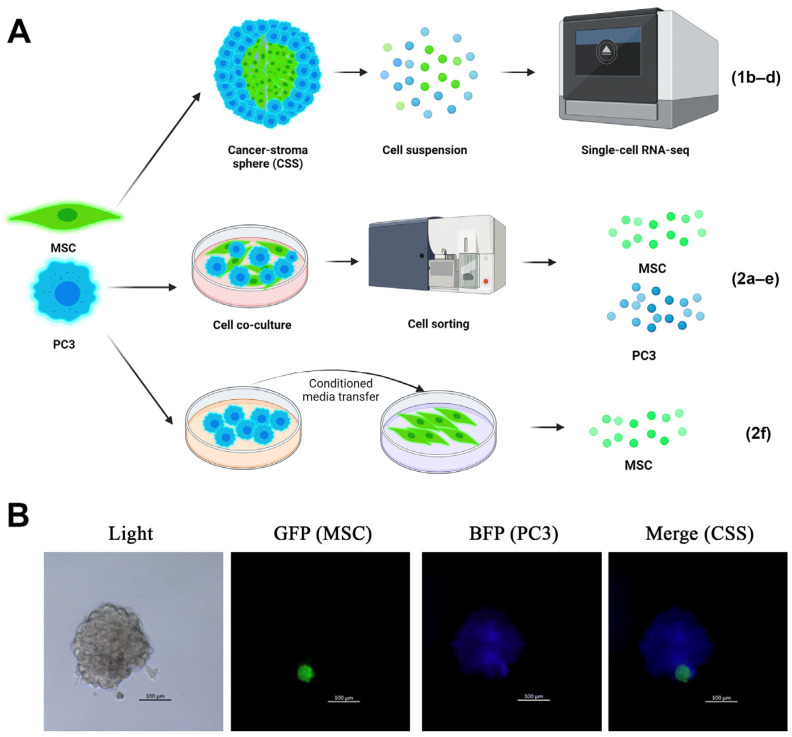

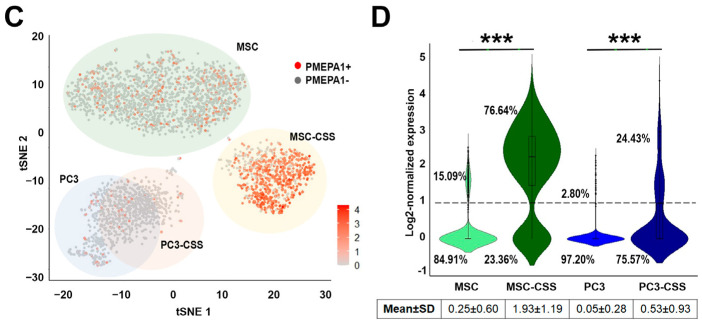
Cell-type-dependent PMEPA1 expression. Schematic representation of experimental design (**A**). PMEPA1 levels in MSCs were studied in long-term cancer–stroma sphere model, short-term PC3 and MSC co-culture and under the PC3-derived conditioned media culture conditions. Representative microphotographs of CSS (**B**). Single-cell RNA sequencing data analysis of cancer-stroma spheres and single PC3 and MSC spheres represented on tSNE plot (**C**). Violin plots show PMEPA1 expression, obtained by single-cell RNA sequencing (**D**). ***—*p* ≤ 0.001.

The hyperexpression of PMEPA1 has previously been demonstrated in TA-MSCs solely using clinical samples. To investigate PMEPA1 expression under in vitro conditions, we employed a CSS model that was previously developed in our laboratory [15]. Prostate cancer PC3 cells and GFP+-immortalized MSCs were cultured in a sphere-forming medium for 11 days, after which the formed spheres underwent enzymatic dissociation and RNA sequencing was conducted on single cells derived from PC3, MCSs, and CSSs (Figure 1A,B). The frequency of heterogeneous sphere formation and the ratio of tumor to stromal cells within CSS have been previously examined in detail in our earlier publication [15,16].

Tumor and mesenchymal cells isolated from CSS further will be denoted as PC3-CSS and MSC-CSS, correspondingly. A total of 1644 MSCs, 732 MSC-CSSs, 786 PC3, and 223 PC3-CSS single-cell transcription profiles were obtained by single-cell sequencing. The MSC population was characterized as DCN+COL1A1+COL1A2 cells, while the PC3 population was identified as MSMP+ cells. Figure 1B presents a t-SNE plot illustrating the close proximity of tumor cells obtained from the PC3 and CSSs. In contrast, MCSs and CSSs form independent cell populations and do not overlap.

Analysis of expression data revealed that initially, 2.80% of PC3 cells and 15.09% of MSCs expressed PMEPA1. Upon co-culture in CSS mode, the expression of PMEPA1 was elevated to 24.43% in PC3 cells and 76.64% in MSCs. Notably, the significant increase in the percentage of MSCs expressing PMEPA1 was accompanied by a rise in the average expression level: 1.93 ± 1.19 in MSC-CSS compared to 0.25 ± 0.60 in MSC (Figure 1C,D). Thus, we have demonstrated that PMEPA1 is hyperexpressed when MSCs co-cultured with prostate cancer, and CSS can serve as a valuable model for cancer–stroma cell interaction studies.

### 2.2. PMEPA1 Is Overexpressed in MSCs During Short-Term Co-Culture with Cancer Cells

**Figure 2:**
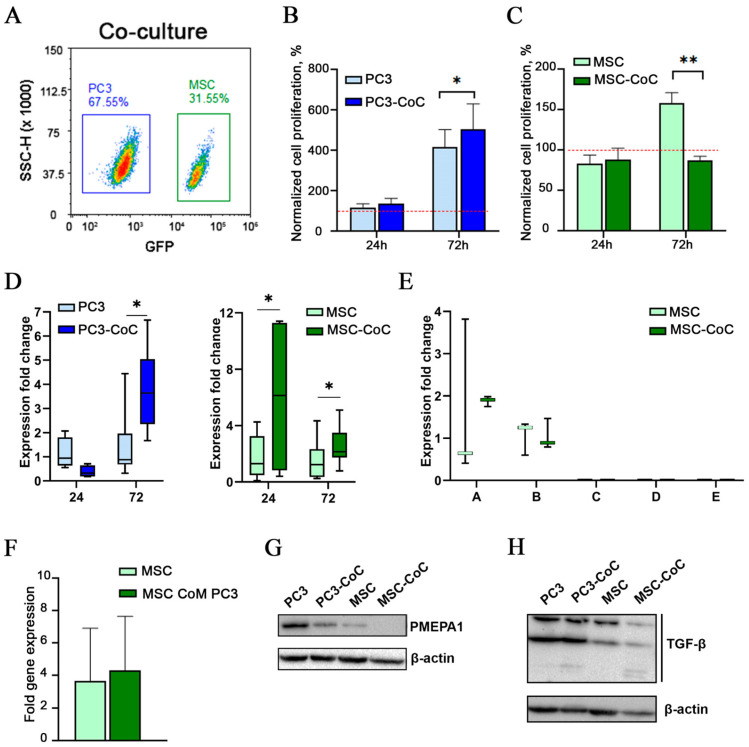
PMEPA1 expression and PMEPA1 isoform study in short term PC3 and MSC co-culture. Representative FACS plot showing gating strategy for PC3 and MCS cell sorting (**A**). Cell count of PC-3 (**B**) and MSCs (**C**) in co-culture conditions The red line represents the number of cells at the time of seeding for cultivation, relative to which cell proliferation was assessed. PMEPA1 expression in PC3 and MSCs, cultured in mono- and co-culture conditions (**D**). Expression of PMEPA1 isoforms in MSCs, cultured in mono- and co-culture conditions (**E**). PMEPA1 expression in MSCs, cultured with PC3-derrived conditional media (CoM) (**F**). Protein levels of PMEPA1 (**G**) and TGF-β (**H**) in PC3 and MSCs, cultured in mono- and co-culture conditions. *—*p* ≤ 0.05, **—*p* ≤ 0.01.

Prolonged co-culture of the cells induces numerous changes in the transcriptome and raises questions regarding secondary alterations. To investigate the expression of PMEPA1 in a short-term culture, we performed a co-culture of PC3 and MSCs under standard adhesive growth conditions. Subsequently, the cells were detached from the plastic and sorted using flow cytometry based on GFP expression at 24 h and 72 h time points (Figure 2A). Sorted cells further were labeled as “PC3-CoC” (co-cultured) and “MSC-CoC” to avoid confusion with sphere-derived cells.

Cell counting revealed that a 24 h co-culture did not significantly affect the proliferative characteristics of the examined cells (Figure 2B,C). However, after 72 h of co-culture, we observed a 5-fold increase from the initial plated cell number in PC3 cell numbers, compared to a 5-fold increase in control PC3 cells (*p* = 0.03). Conversely, MSCs demonstrated a diminished proliferation when co-cultured with tumor cells, with proliferation levels decreasing to 87.96 ± 4.70%, compared to 158.71 ± 12.56% in MSC monoculture (*p* = 0.008).

Furthermore, the level of PMEPA1 mRNA was significantly elevated in MSCs cultured for 24 h with tumor cells compared to those maintained in a MSC monoculture (Figure 2D, *p* < 0.05). Notably, the co-culture of cells for 72 h resulted in a significant increase in PMEPA1 expression in both mesenchymal and tumor cells (Figure 2D, *p* < 0.05).

To determine the isoforms of PMEPA1 that are expressed in MSCs, we conducted an expression analysis utilizing primers specific to each of the five gene isoforms. Expression of PMEPA1a and PMEPA1b isoforms has been detected in MSCs (Figure 2E).

To assess the role of secreted factors from tumor cells in enhancing PMEPA1 expression levels, we performed experiments involving the transfer of conditioned media from tumor cells to mesenchymal stem cells, followed by a 24 h culture period. Notably, we did not observe a significant change in PMEPA1 levels in MSCs exposed to conditioned media from tumor cells, suggesting a potential role for autocrine regulation within MSCs, possibly involving TGF-β secreted by MSCs (Figure 2F).

To determine TGF-β levels, we conducted a Western blot analysis of cell lysates from PC3, PC3-CoC, MSC, and MSC-CoC cells. Despite the observed increase in PMEPA1 mRNA levels in both tumor and stromal cells following 72 h of co-culture, we identified a decrease in the target protein levels (Figure 2G). Based on the prevalence of PMEPA1a mRNA isoform in the MSCs and the size of the band, we suggest that PMEPA1a protein level has been detected by Western blot in the MSC sphere but undetected in MSC-CoC. Analysis of TGF-β revealed three bands at approximately 44 kDa, 35 kDa, and 25 kDa. The band with the lowest molecular weight was present in the MSC-CoC sample but was undetectable in MSCs alone.

### 2.3. TGF-β3 Is Overexpressed in PMEPA1+ CSS-Derived Mesenchymal Cells

**Figure 3:**
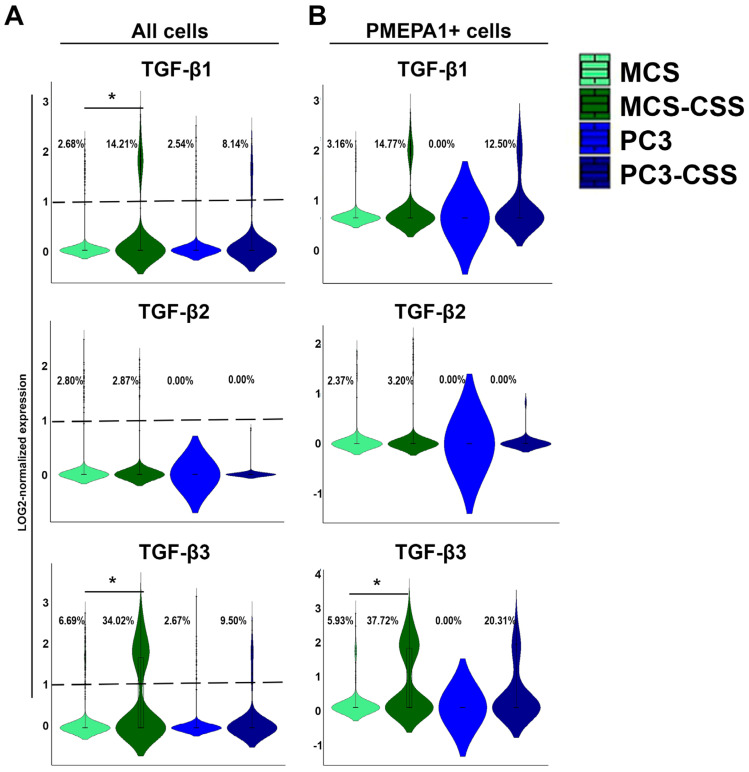
Expression of PMEPA1-regulating genes in PC3, MSC spheres, and CSS: TGF-β1, TGF-β2 and TGF-β3 mRNA levels shown for all cells (**A**) and PMEPA1-positive cell populations (**B**). *—*p* ≤ 0.05.

To further investigate interplay between TGF-β and PMEPA1, we went back to the single-cell sequencing data of CSS. As expression of PMEPA1 in androgen-independent tumor cells is primarily regulated by TGF-β, we have compared mRNA levels of TGF-β1, TGF-β2 and TGF-β3 in MSCs and PMEPA1-expressing MSCs (Figure 3).

Our analysis of TGFB transcription revealed significant changes in the levels of TGF-β1 and TGF-β3 within MSCs present in the CSS, but only TGF-β3 level has been significantly elevated in PMEPA1+ cells (Figure 3A). TGF-β3 expression was detected in 37.7% of MSC-CSS as compared to 5.93% of MSCs (Figure 3B).

## 3. Discussion

In the present study, we demonstrate that the mRNA expression levels of PMEPA1 are altered not only in cancer cells but also in mesenchymal stem cells during in vitro PC3 and MSC co-culture. Utilized heterogeneous spheres composed of tumor and stromal cells (CSS) as our model system (Figure 1B) allowed to investigate the reciprocal influence of two cell factors on the cell proliferation and expression profiles [15]. Our findings indicate that the number of PMEPA1-expressing cells, as well as the mean expression level of PMEPA1, increase in MSCs, cultured within CSS (Figure 1C,D). Furthermore, changes in PMEPA1 expression arise as an early event of cell co-culture and can be detected at the 24 h time point (Figure 2D). PMEPA1a and PMEPA1b isoforms are expressed in MSCs, indicating involvement of both TGF-β and AR-pathways in MSC control. PMEPA1 isoforms have not been described in MSC before. An increase in PMEPA1 expressions is accomplished with a diminished proliferation of MSCs cultured within the CSS (Figure 2C).

In the context of the established interaction between PMEPA1 and the TGF- β and AR signaling pathways [17], for AR-negative PC3 cells only TGF-beta pathway remains in the focus [18]. Cancer spheres can reach several hundred microns in diameter and develop hypoxia in the center of the sphere. The activation of the TGF-beta signaling pathway, which regulates protease production, leads to subsequent tissue structural degradation and the release of tumor cells, which is an anticipated consequence. Moreover, previous studies have demonstrated that prolonged culture of MSCs and breast cancer cells can activate the TGF-beta signaling pathway [19]. Therefore, we selected a long-term co-culture model of tumor and stromal cells within a CSS to study PMEPA1 levels in MSCs. This model does not require the artificial addition of growth factors and more accurately replicates in vivo tumor growth [15].

Following an 11-day culture period, we observed significant overexpression of TGF-β1 and TGF-β3 isoforms in MSCs grown under CSS conditions (Figure 3A). The increase in PMEPA1 expression was statistically significant in both tumor and mesenchymal cells, with more pronounced differences observed in mesenchymal cells: 24.43% of PC3 cells and 76.64% of MSCs exhibited increased expression. Previous research has indicated that the addition of TGF-β to the culture medium of tumor cells alters PMEPA1 expression within just two hours [7]. In our study, where TGF-β was not artificially added but was produced by the cells themselves, we demonstrated that PMEPA1 expression significantly changed in mesenchymal cells after 24 h. In contrast, this effect was only observed in tumor cells after 72 h.

Despite the observed elevation of TGF-β and PMEPA1 expressions at the mRNA level, protein analyses indicated a decrease in their levels in MSCs co-cultured with cancer cells for 72 h (Figure 2H). This discrepancy raises important questions regarding the dynamics of TGF-β and PMEPA1 in the cellular environment. One possible explanation for the reduced protein levels is the release of TGF-β during cell–cell interactions, which can lead to its utilization by neighboring cells [20].

However, the concurrent “disappearance” of PMEPA1 as a protein, despite high gene expression and the presence of its transcripts in MSCs, complicates this finding. This situation presents a significant challenge in understanding the degradation mechanisms of PMEPA1, whether at the mRNA or protein level. One potential pathway for protein degradation could involve exocytosis, similar to the release of extracellular vesicles loaded with PMEPA1 mRNA from glioblastoma stem cells [21]. Further investigation is necessary to elucidate these mechanisms and their implications for cellular communication and signaling in the tumor microenvironment.

The tumor stroma is highly dynamic and heterogeneous. Studies have demonstrated that over 60% of tumors exhibit a high tumor–stroma ratio, indicating that more than half of the cellular composition within a tumor consists of stromal cells [22]. This observation opens up the possibility of targeting stromal cells for cancer treatment. Several clinical trials have focused on targeting tumor-associated immune cells, vascular cells, and fibroblasts (reviewed in [23]). PMEPA1 represents a promising target for stromal cell intervention, given its low basal expression in normal cells and its significant upregulation within the tumor microenvironment. The existence of five isoforms, along with their tissue-specific expression patterns, may facilitate the development of more precise strategies to inhibit the growth of pro-tumor microenvironment cells. However, we recognize that further investigation into PMEPA1 expression in normal cells, as well as its induced expression across various stromal cell populations influenced by cancer cells, is essential for the formulation of effective treatment strategies.

## 4. Materials and Methods

### 4.1. Cell Culture

Immortalized mesenchymal stem cells (MSCs) expressing the green fluorescent protein (GFP) were previously generated in our laboratory through the hyperexpression of the hTERT-GFP genes and suppression of p53 expression [24]. The PC3-BFP cell line was obtained via lentiviral transduction with a blue fluorescent protein [15]. Both cell lines were cultured in RPMI-1640 medium (Paneco, Moscow, Russia) supplemented with 10% fetal bovine serum (FBS, Hyclone, Logan, UT, USA), 2 mM L-glutamine (Paneco, Moscow, Russia), and 50 units/mL penicillin and 50 µg/mL streptomycin (Paneco, Moscow, Russia) then indicated as complete RPMI-1640 medium. Cultures were maintained at 37 °C in a humidified atmosphere containing 5% CO_2_. The authenticity of the cell lines confirmed by short tandem repeat DNA genotyping by GORDIZ Ltd. (Moscow, Russia).

### 4.2. Sphere Formation Assay

Spheres were generated in 35 mm non-adherent Petri dishes using a sphere formation medium composed of DMEM/F-12 (Paneco, Moscow, Russia), 2 × B27 (Paneco, Moscow, Russia), 40 ng/mL of fibroblast growth factors 2 (FGF2), and 40 ng/mL of epidermal growth factor (EGF) (Sci-store, Moscow, Russia), 2 mM L-glutamine, 50 units/mL penicillin, and 50 µg/mL streptomycin (Paneco, Moscow, Russia) (SFA medium). The initial cell density was set at 1000 cells/mL for PC3 cells and 5000 cells/mL for MSCs. To create heterogeneous spheroids, cells were mixed as 1000 PC3 cells and 5000 MSCs per ml of medium. Spheres were counted after a 7-day culture period using a Zeiss STEMI stereomicroscope (Carl Zeiss, Oberkochen, Germany).

### 4.3. Single-Cell Sequencing

For single-cell sequencing, a cell suspension was prepared. Spheres were harvested by centrifugation at 400× *g* for 5 min. To dissociate the cells, trypsin–EDTA (0.25%, Paneco, Moscow, Russia) was added, and the mixture was incubated at 37 °C for 10 min. Following trypsinization, complete RPMI-1640 medium (Paneco, Moscow, Russia) was added to neutralize trypsin activity. The cells were then centrifuged and resuspended for further analysis.

Single-cell RNA libraries were prepared using the Chromium Controller system and the Chromium Single Cell 3′ Reagent Kit (v3.1) (10× Genomics, Pleasanton, CA, USA). Cell suspensions were loaded onto a 10× Genomics Single Cell fluidics chip targeting a recovery of approximately 2000 cells. Cells were partitioned into single-cell-containing Gel Beads in Emulsion (GEMs), and single-cell libraries were constructed following the manufacturer’s instructions. The resulting libraries were sequenced on the NextSeq500 system (Illumina, San Diego, CA, USA) with a recommended sequencing run depth of approximately 20,000 read pairs per cell.

### 4.4. Cell Co-Culture

For the co-culture experiments, a preliminary mixing of 15,000 PC3 cells/cm^2^ and 45,000 MSCs/cm^2^ was performed. The cells were cultured in complete RPMI-1640 medium. The cultures were maintained at 37 °C in a humidified atmosphere containing 5% CO_2_.

### 4.5. Cell Sorting

After 24 and 72 h of co-cultivation, the cells were dissociated using trypsin–EDTA (0.25%, Paneco, Moscow, Russia). Following dissociation, the cells were pelleted and resuspended in DPBS. Subsequently, GFP-positive MSCs and GFP-negative PC3 cells were sorted using a FACSAria III cell sorter (BD Biosciences, Franklin Lakes, NJ, USA).

### 4.6. Conditioned Media Transfer

To assess the impact of tumor-cell-conditioned media on MSCs, both cell types were plated at a density of 60,000 cells/cm^2^ in complete RPMI-1640 medium. After 24 h, the conditioned media from tumor cells was collected and filtered through a PES filter with a pore size of 0.45 µm. The conditioned media from MSCs was removed, cells were washed with DPBS. The filtered tumor-cell-conditioned media was then transferred to the MSC cultures. Following 24 h of incubation, the MSCs were collected for further analysis.

### 4.7. Quantitative Reverse Transcription Polymerase Chain Reaction (RT-qPCR)

Total RNA was extracted using the Quick-RNA Miniprep Kit (Zymo Research, Irvine, CA, USA) according to the manufacturer’s protocol. Complementary DNA (cDNA) was synthesized using the RevertAid First Strand cDNA Synthesis Kit (Thermo Fisher Scientific, Waltham, MA, USA) on a C1000 Touch Thermal Cycler (Bio-Rad, Hercules, CA, USA). RT-qPCR was performed on a CFX Connect Real-Time PCR Detection System (Bio-Rad, Hercules, CA, USA) using the 5X qPCRmix-HS SYBR master mix (Evrogen, Moscow, Russia). The GAPDH gene was employed for normalization of each sample. Differences in gene expression were assessed by comparing ΔCT values using the ΔΔCt method. Fold change gene expression was calculated as 2^−ΔΔCt^. The primers utilized for qRT-PCR analysis were described in [8,17] are listed in Table 1.

### 4.8. Single-Cell RNA Statistical Analysis

The initial processing of FASTQ files has been performed using the Cell Ranger software environment v 7.1.0., specifically designed for single-cell sequencing analysis. Quality control, analysis, and exploration of single-cell RNA sequencing data were performed utilizing the Seurat package (version 5.2.1). Data normalization was achieved through the “LogNormalize” method, followed by principal component analysis (PCA) for dimensionality reduction and cluster identification. For creating plots ggplot2 v 3.5.2 and patchwork libraries v 1.3.0 were applied.

### 4.9. Western Blot Analysis

PC3 and MSCs were co-cultured for 72 h and FACS-sorted as described above. Cells were lysed in RIPA buffer (ServiceBio, Wuhan, China) containing 1× protease inhibitor (Thermo Fisher Scientific, USA). Protein concentration was measured using Pierce BCA Protein Assay Kit (Thermo Fisher Scientific, USA). Proteins (20 μg) were separated by 10% SDS-PAGE, transferred into PVDF membrane and detected by primary antibodies for PMEPA1 (Affinity Bioscience, #DF3391, Shanghai, China) and TGF-β1 (Affinity Bioscience, #BF0334, China). The HRP-conjugated antibody to β-actin (GenScript, Piscataway, NJ, USA) was used for the detection of β-actin as loading control. Chemiluminescence signal was developed with Clarity Western ECL Substrate kit and registered using ChemiDoc XRS Plus (Bio-Rad Laboratories, Inc., USA).

### 4.10. Statistical Analysis

Differential gene expressions were assessed by Student’s *t*-test analysis that was adjusted for multiple testing using the Benjamini–Hochberg procedure. To evaluate differences in median values across multiple groups, the Kruskal–Wallis one-way analysis of variance on ranks was conducted, followed by multiple comparison procedures using Dunn’s method.

## 5. Conclusions

The TGF-β signaling pathway is one of the critical pathways regulating tumor metastasis and disease aggressiveness. Notably, the primary producers of TGF-β are often not the tumor cells themselves but rather the stromal cells within the tumor microenvironment. PMEPA1 functions both as a direct target gene and as a regulator that restrains the activity of the TGF-β signaling pathway. Its expression in mesenchymal stem cells, along with the modulation of its expression levels by tumor cells at short-term culture, suggests that the role of PMEPA1 in regulating the tumor microenvironment may be underestimated. Further research is essential to fully understand PMEPA1’s implications in cancer biology and how it might influence tumor microenvironment.

## Figures and Tables

**Table 1:** RT-qPCR primers.

Title	Sequence
hPMEPA1 total_RT-F	CTGAGCCACTACAAGCTGTCTG
hPMEPA1 total_RT-R	GGATTCCGTTGCCTGACACTGT
hPMEPA1a-RT-F	GCAACTGCAAACGCTCTTTGT
hPMEPA1a-RT-R	GGACCGTGCAGACAGCTTGTA
hPMEPA1b-RT-F	CATCATCCCCGAGCTGCT
hPMEPA1b-RT-R	TGATCTGAACAAACTCCAGCTCC
hPMEPA1c-RT-F	GGATGAATTCGCTCTGGTCTAG
h-PMEPA1cd-RT-R	ACCACCATCACCATCATCAC
hPMEPA1d-RT-F	ACAGGCGAAAAGTCAAAATGC
hPMEPA1e-RT-F	CTTCCCCGTGTGCAAGAG
hPMEPA1e-RT-R	CTGGATCCTCAGCCACTG

## Data Availability

The original contributions presented in this study are included in the article. Further inquiries can be directed to the corresponding author.

## References

[1] Itoh S., Thorikay M., Kowanetz M., Moustakas A., Itoh F., Heldin C.-H., Dijke P.T. (2003). Elucidation of Smad requirement in transforming growth factor-beta type I receptor-induced responses. J. Biol. Chem..

[2] Xu L.L., Shanmugam N., Segawa T., Sesterhenn I.A., McLeod D.G., Moul J.W., Srivastava S. (2000). A novel androgen-regulated gene, PMEPA1, located on chromosome 20q13 exhibits high level expression in prostate. Genomics.

[3] Brunschwig E.B., Wilson K., Mack D., Dawson D., Lawrence E., Willson J.K.V., Lu S., Nosrati A., Rerko R.M., Swinler S. (2003). PMEPA1, a transforming growth factor-beta-induced marker of terminal colonocyte differentiation whose expression is maintained in primary and metastatic colon cancer. Cancer Res..

[4] Watanabe Y., Itoh S., Goto T., Ohnishi E., Inamitsu M., Itoh F., Satoh K., Wiercinska E., Yang W., Shi L. (2010). TMEPAI, a transmembrane TGF-beta-inducible protein, sequesters Smad proteins from active participation in TGF-beta signaling. Mol. Cell.

[5] Sharad S., Dobi A., Srivastava S., Srinivasan A., Li H. (2020). PMEPA1 Gene Isoforms: A Potential Biomarker and Therapeutic Target in Prostate Cancer. Biomolecules.

[6] Piqué-Gili M., Andreu-Oller C., Mesropian A., Esteban-Fabró R., Bárcena-Varela M., de Galarreta M.R., Montironi C., Martinez-Quetglas I., Cappuyns S., Peix J. (2024). Oncogenic role of PMEPA1 and its association with immune exhaustion and TGF-beta activation in HCC. JHEP Rep..

[7] Fournier P.G., Juárez P., Jiang G., Clines G.A., Niewolna M., Kim H.S., Walton H.W., Peng X.H., Liu Y., Mohammad K.S. (2015). The TGF-beta Signaling Regulator PMEPA1 Suppresses Prostate Cancer Metastases to Bone. Cancer Cell.

[8] Sharad S., Dillman A.A., Sztupinszki Z.M., Szallasi Z., Rosner I., Cullen J., Srivastava S., Srinivasan A., Li H. (2020). Characterization of unique PMEPA1 gene splice variants (isoforms d and e) from RNA Seq profiling provides novel insights into prognostic evaluation of prostate cancer. Oncotarget.

[9] Yu R., Han H., Chu S., Qin L., Du M., Ma Y., Wang Y., Jiang W., Song Y., Zou Y. (2025). Cullin 4B-RING E3 ligase negatively regulates the immunosuppressive capacity of mesenchymal stem cells by suppressing iNOS. Cell Death Differ..

[10] Raghav P.K., Mann Z. (2021). Cancer stem cells targets and combined therapies to prevent cancer recurrence. Life Sci..

[11] Lourenco S., Maughan E., Janes S. (2015). MSC homing & immunomodulatory properties in cancer therapies: Searching for the perfect balance. Cell Gene Ther. Insights.

[12] Fregni G., Quinodoz M., Möller E., Vuille J., Galland S., Fusco C., Martin P., Letovanec I., Provero P., Rivolta C. (2018). Reciprocal modulation of mesenchymal stem cells and tumor cells promotes lung cancer metastasis. eBioMedicine.

[13] Brennen W.N., Chen S., Denmeade S.R., Isaacs J.T. (2013). Quantification of Mesenchymal Stem Cells (MSCs) at sites of human prostate cancer. Oncotarget.

[14] Shahar T., Rozovski U., Hess K.R., Hossain A., Gumin J., Gao F., Fuller G.N., Goodman L., Sulman E.P., Lang F.F. (2017). Percentage of mesenchymal stem cells in high-grade glioma tumor samples correlates with patient survival. Neuro Oncol..

[15] Rakhmatullina A.R., Zolotykh M.A., Filina Y.V., Mingaleeva R.N., Sagdeeva A.R., Boulygina E.A., Gafurbaeva D.U., Bulatov E.R., Rizvanov A.A., Miftakhova R.R. (2024). Development of a novel prostate Cancer-Stroma Sphere (CSS) model for In Vitro tumor microenvironment studies. Transl. Oncol..

[16] Rakhmatullina A.R., Zolotykh M.A., Filina Y.V., Valiullina A.K., Zmievskaya E.A., Gafurbaeva D.U., Sagdeeva A.R., Bulatov E.R., Rizvanov A.A., Miftakhova R.R. (2024). Multicellular Cancer-Stroma Spheres (CSS) for In Vitro Assessment of CAR-T Cell-Associated Toxicity. Cells.

[17] Sharad S., Sztupinszki Z.M., Chen Y., Kuo C., Ravindranath L., Szallasi Z., Petrovics G., Sreenath T.L., Dobi A., Rosner I.L. (2019). Analysis of PMEPA1 Isoforms (a and b) as Selective Inhibitors of Androgen and TGF-beta Signaling Reveals Distinct Biological and Prognostic Features in Prostate Cancer. Cancers.

[18] van Bokhoven A., Varella-Garcia M., Korch C., Johannes W.U., Smith E.E., Miller H.L., Nordeen S.K., Miller G.J., Lucia M.S. (2003). Molecular characterization of human prostate carcinoma cell lines. Prostate.

[19] Melzer C., von der Ohe J., Otterbein H., Ungefroren H., Hass R. (2019). Changes in uPA, PAI-1, and TGF-beta Production during Breast Cancer Cell Interaction with Human Mesenchymal Stroma/Stem-Like Cells (MSC). Int. J. Mol. Sci..

[20] Peterson A.J., O’connor M.B. (2018). Lean on Me: Cell-Cell Interactions Release TGF-beta for Local Consumption Only. Cell.

[21] Castellani G., Buccarelli M., D’aLessandris Q.G., Ilari R., Cappannini A., Pedini F., Boe A., Lulli V., Parolini I., Giannetti S. (2024). Extracellular vesicles produced by irradiated endothelial or Glioblastoma stem cells promote tumor growth and vascularization modulating tumor microenvironment. Cancer Cell Int..

[22] Pyo J.-S., Kim N.Y., Min K.-W., Kang D.-W. (2023). Significance of Tumor-Stroma Ratio (TSR) in Predicting Outcomes of Malignant Tumors. Medicina.

[23] Kozlova N., Grossman J.E., Iwanicki M.P., Muranen T. (2020). The Interplay of the Extracellular Matrix and Stromal Cells as a Drug Target in Stroma-Rich Cancers. Trends Pharmacol. Sci..

[24] Rakhmatullina A.R., Mingaleeva R.N., Gafurbaeva D.U., Glazunova O.N., Sagdeeva A.R., Bulatov E.R., Rizvanov A.A., Miftakhova R.R. (2023). Adipose-Derived Mesenchymal Stem Cell (MSC) Immortalization by Modulation of hTERT and TP53 Expression Levels. J. Pers. Med..

